# Short- and long-term outcomes of patients with minor stroke and nonvalvular atrial fibrillation

**DOI:** 10.1186/s12883-023-03457-3

**Published:** 2023-11-20

**Authors:** Chunmiao Duan, Shang Wang, Yunyun Xiong, Hong qiu Gu, Kaixuan Yang, Xing-Quan Zhao, Xia Meng, Yongjun Wang

**Affiliations:** 1 Vascular Neurology, Department of Neurology, Beijing Tiantan Hospital, Capital Medical University, Beijing, China; 2 China National Clinical Research Center for Neurological Diseases, Beijing, China; 3 Department of Neurology, Beijing Daxing Teaching Hospital, Capital Medical University, Beijing, China; 4 Neurocardiology Center, Department of Neurology, Beijing Tiantan Hospital, Capital Medical University, Beijing, China; 5 Chinese Institute for Brain Research, Beijing, China; 6 National Center for Healthcare Quality Management in Neurological Diseases, Beijing, China; 7 Center for Stroke, Beijing Institute for Brain Disorders, Beijing, China

**Keywords:** Stroke, Atrial Fibrillation, Mortality, recurrent Stroke

## Abstract

**Background and purpose:**

Nonvalvular atrial fibrillation (NVAF) is a risk factor for stroke. This study was undertaken to determine the influence of NVAF on the mortality and recurrent stroke after a minor stroke event.

**Methods:**

Data were derived from the Third China National Stroke Registry (CNSR-III) which enrolled 15,166 subjects during August 2015 through March 2018 in China. Patients with minor stroke (NIHSS ≤ 5) within 24 h after onset were included. Clinical outcomes including all-cause mortality, cardiovascular death, recurrent ischemic stroke, and recurrent hemorrhagic stroke were collected. The Cox proportional hazards models were used to determine the association between NVAF and clinical outcomes.

**Results:**

A total of 4,753 patients were included in our study. Of them, 222 patients had NVAF (4.7%) (mean age, 71.1 years) and 4,531 patients were without AF (95.3%) (mean age, 61.4 years). NVAF was associated with 12-month cardiovascular mortality in both univariate (hazards ratio [HR], 4.13; 95% confidence interval [CI], 1.84 to 9.31; *P* < 0.001) and multivariate analyses (HR, 4.66; 95% CI, 1.79 to 12.15; *P* = 0.001). There was no difference in the in-hospital ischemic stroke recurrence rate between the two groups (HR, 0.45 [95% CI, 0.19 to 1.05] *P* = 0.07 at discharge). However, patients with NVAF had a lower rate of recurrent ischemic stroke at medium- (3 months and 6 months) and long-term (12 months) follow-up (HR, 0.33 [95% CI, 0.16 to 0.68] *P* = 0.003 at 3 months; 0.49 [95% CI, 0.27 to 0.89] *P* = 0.02 at 6 months; 0.55 [95% CI, 0.32 to 0.94] *P* = 0.03 at 12 months, respectively) compared with those without. There was no difference in all-cause mortality and hemorrhagic stroke between the two groups during follow-up.

**Conclusions:**

Minor stroke patients with NVAF were at higher risk of cardiovascular death but had a lower rate of recurrent ischemic stroke compared to those without during the subsequent year after stroke event. A more accurate stroke risk prediction model for NVAF is warranted for optimal patient care strategies.

**Supplementary Information:**

The online version contains supplementary material available at 10.1186/s12883-023-03457-3.

## Background

Atrial fibrillation (AF) is a major risk factor for stroke, resulting in a 3- to 5-fold risk of stroke occurrence and a 2-fold risk of mortality [1, 2]. The Global Burden of Disease Study 2020 estimated a worldwide prevalence of AF around 50 million individuals and deaths for AF around 0.33 million in 2020 [3]. The burden of AF is increasing with ageing population. In China, prevalent AF in 2050 will be about 8.3 million among individuals older than 60 years [4].

About 20% ischemic strokes is associated with cardioembolic source [5], and 80% cardioembolic stroke is attributable to AF. Ischemic strokes related to AF are prone to present with large-territory infarct and a high incidence of substantial disability and mortality [6, 7]. Nevertheless, patients with acute ischemic stroke and AF might present with low NIHSS scores, partly due to good collateral status and blood clots self-melting. Moreover, larger randomized controlled trials of minor stroke including CHANCE (Clopidogrel in High-Risk Patients with Acute Nondisabling Cerebrovascular Events) and POINT (Platelet-Oriented Inhibition in New TIA and Minor Ischemic Stroke) excluded cardioembolic stroke [8, 9]. Up to now, there is limited evidence of outcomes specified for acute minor stroke with nonvalvular AF (NVAF).

We aimed to investigate the short- and long-term mortality and recurrent stroke of patients with acute minor ischemic stroke and NVAF within 24 h from onset. We hypothesized that acute minor ischemic stroke patients with NVAF had worse outcomes.

## Methods

The Third China National Stroke Registry (CNSR-III) was a nationwide, hospital-based, prospective registry in China, as described elsewhere [10]. CNSR-III enrolled patients aged 18 years or older with a primary diagnosis of acute ischemic stroke (AIS) or transient ischemic attack (TIA) within 7 days between August 2015 and March 2018. Informed consent from patients or their next of kin was obtained. The data in a uniform format through electronic data capture system were collected by trained research coordinators and checked automatically. All image data were collected and analyzed centrally at institutional Neuroimaging Center of Excellence. The study was approved by the ethics committee of all participating study centers.

### Study population

A total of 15,166 consecutive patients were included from 201 hospitals that cover 22 provinces and four municipalities in China in CNSR-III. We derived the patients fulfilling the following inclusion criteria: [1] minor ischemic stroke: NIHSS ≤ 5; [2] time window: onset to door time < 24 h. The exclusion criteria were as follows: [1] admission diagnosis of transient ischemic attack (TIA); [2] premorbid slight disability, defined as modified Rankin Scale (mRS) ≥ 2 (mRS, range 0–6, with lower scores indicating better functional status); [3] valvular AF. AF was identified by prior medical history, electrocardiography (ECG) and/or 24-hour ECG monitoring during admission or hospitalization. Patients were divided into acute minor stroke patients with NVAF and those without AF groups.

### Data collection and definitions

We extracted the following variables: demographics (including age and sex), medical history (including current smoking, body mass index [BMI, calculated as weight in kilograms divided by height in meters squared], hypertension, diabetes mellitus, dyslipidemia, coronary artery disease, heart failure, previous stroke/TIA, peripheral vascular disease [PVD]), medication history (including antiplatelet and anticoagulant agents), baseline random blood glucose, baseline systolic blood pressure, baseline diastolic blood pressure, initial neurological status (measured by NIHSS, range 0 to 42, with higher scores indicating severe stroke), CHA_2_DS_2_-VASc score (congestive heart failure, hypertension, age ≥ 75 years, diabetes, stroke/transient ischemic attack/thromboembolism, vascular disease, age 65 to 74 years, and sex category) [11], onset to door time, length of stay, in-hospital medications and reperfusion strategy. Etiology classification of ischemic stroke was based on an expanded version of the TOAST (Trial of Org 10,172 in Acute Stroke Treatment) criteria [12].

### Blood sample collection

Elbow venous blood was drawn within 24 h of admission. Plasma specimens were extracted, aliquoted and transported through cold chain to the institutional central clinical laboratory and stored at -80℃. No freezing and thawing circle occurred before test. Serum creatinine, alanine aminotransferase (ALT), and random blood glucose were tested using Hitachi 7600 automatic biochemistry analyzer (Hitachi, Tokyo, Japan), which was described before [13]. All measurements were performed centrally and blindly.

### Clinical outcomes

The primary outcome was cardiovascular death, which was caused by ischemic stroke, intracranial hemorrhage, myocardial infarction, sudden cardiac death, heart failure, pulmonary embolism, aortic aneurysm rupture and peripheral arterial disease. The secondary outcomes included all-cause death, recurrent ischemic stroke, and recurrent hemorrhagic stroke. All-cause mortality was defined as death from any cause. Ischemic stroke was defined as a new symptomatic neurologic deterioration and was identified by neuroimaging evidence of ischemic injury even if the duration of symptoms was less than 24 h. Hemorrhagic stroke was defined as acute extravasation of blood into the brain parenchyma, ventricle, and subarachnoid and was accompanied by neuroimaging evidence of hemorrhage.

### Statistical analysis

The data were tested for normal distribution using the Kolmogorov-Smirnov test. Continuous variables were expressed as mean with standard deviation (SD) or as median with interquartile range (IQR), as appropriate, and differences were assessed using independent 2-sample 𝑡 test if normally distributed or Mann-Whitney *U* test. Categorical data were presented as frequencies with percentages, and the 𝝌^2^ test was used to compare the distributions between groups. To identify the associations of AF with recurrent stroke, and death, the Kaplan-Meier survival curves and log-rank test were performed for the univariate analysis. The Cox proportional hazards models were used for the multivariate analysis, adjusting for covariates that were significant at the *P* value of < 0.1 level in the univariate analysis. Hazard ratios (HRs) were presented with 95% confidence interval (CI). Two-sided *P* < 0.05 was deemed statistically significant. Statistical analyses were conducted using the SAS, version 9.4, software (SAS Institute, Cary, NC, USA).

## Results

### Patient flowchart

**Fig. 1:**
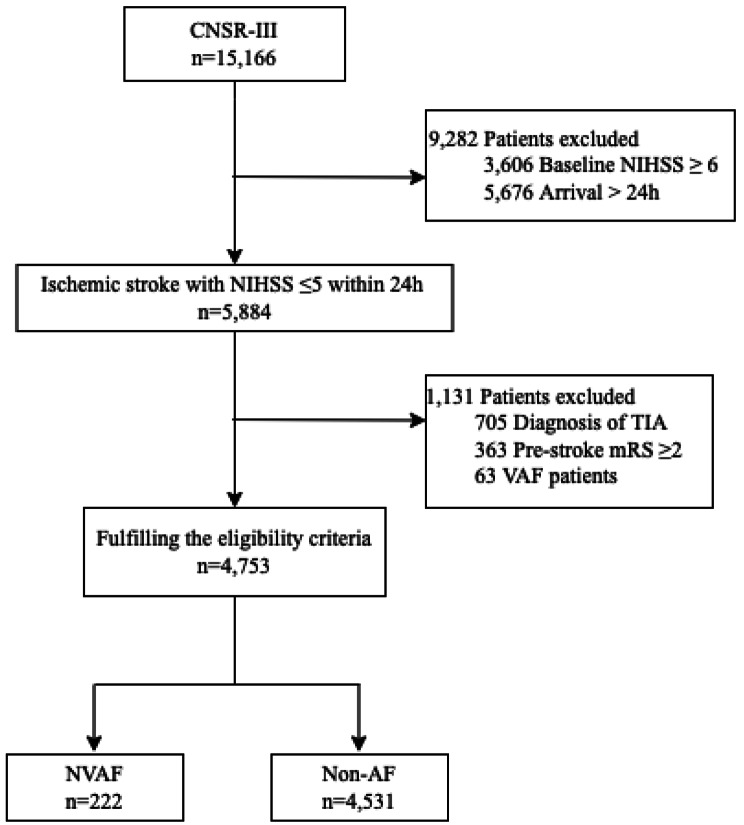
Flowchart CNSR-III indicates the Third China National Stroke Registry; NIHSS, National Institutes of Health Stroke Scale; TIA, transient ischemic attack; mRS, modified Rankin Scale; VAF, valvular atrial fibrillation; NVAF, nonvalvular atrial fibrillation

A total of 4,753 patients with minor ischemic stroke (NIHSS ≤ 5) within 24 h of symptom onset were included and the flowchart (Fig. 1) depicted the reasons for patients exclusion, including baseline NHISS ≥ 6 (n = 3,606), onset to door time > 24 h (n = 5,676), admission diagnosis of TIA (n = 705), premorbid mRS ≥ 2 (n = 363), and valvular AF (n = 63). Finally, 222 patients (4.7%) with NVAF and 4531 patients (95.3%) without AF were selected for subsequent analysis.

### Baseline characteristics

**Fig. 2:**
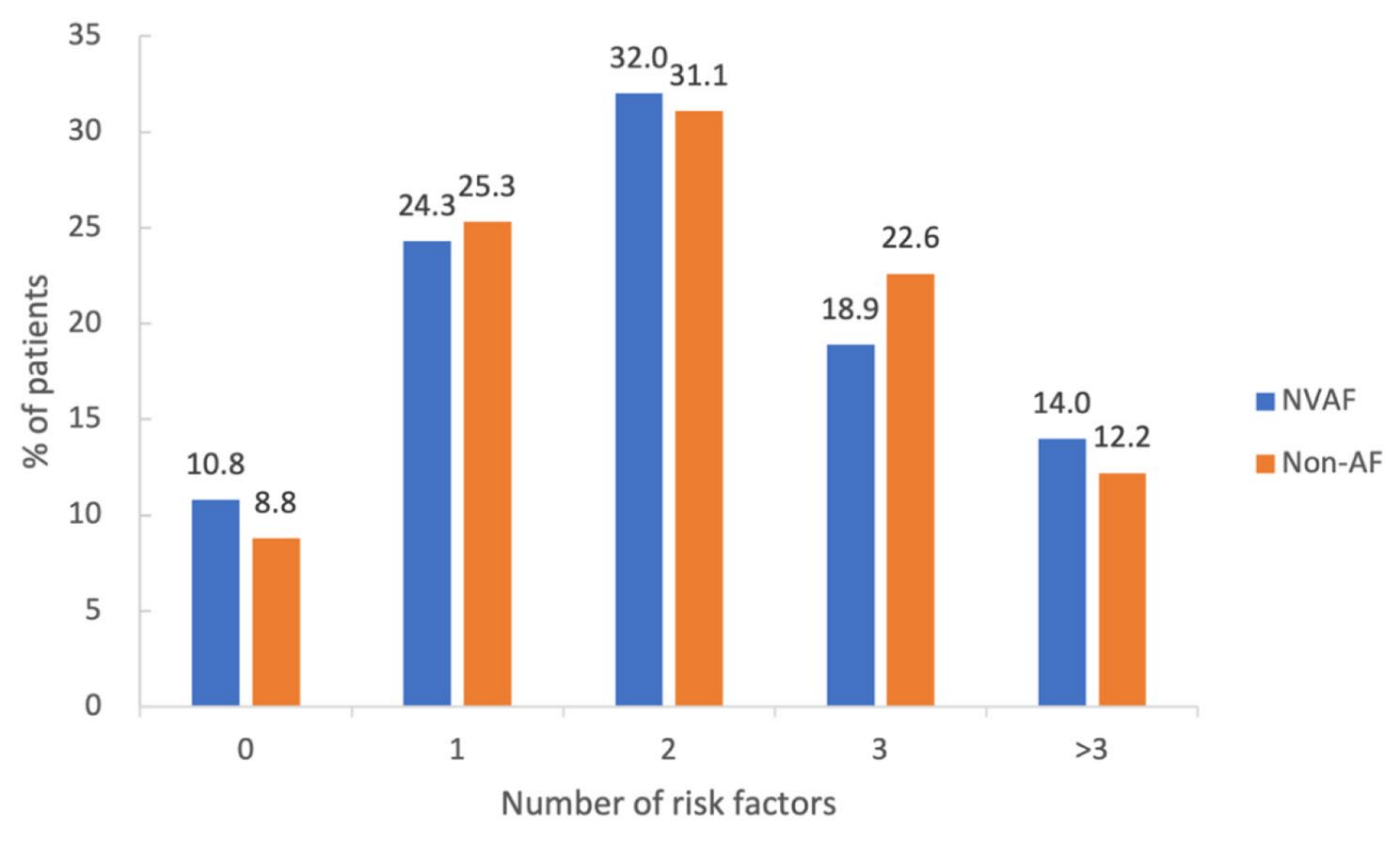
Number and distribution of vascular risk factors based on atrial fibrillation Vascular risk factors include current smoking, hypertension, hyperlipidemia, diabetes mellitus, coronary artery disease, heart failure, prior stroke/transient ischemic attack, peripheral vessel disease, and body mass index ≥ 25 kg/m^2^. NVAF indicates nonvalvular atrial fibrillation

The baseline characteristics of the 4753 patients are described in Table 1. Mean age of the enrolled patients was 61.9 ± 11.1 years, and 29.7% of them were women. The medium onset to door time was 5.9 h (interquartile range [IQR], 2.4–12.4). Of them, 1513 patients (31.8%) were within 3-hour time window, 503 (10.6%) within 3-4.5 h, and 2737 (57.6%) beyond 4.5 h. The median length of hospitalization was 12 days. Among 222 patients with minor stroke and NVAF, 124 patients had prior NVAF and 98 were diagnosed with NVAF by admission ECG (54 cases) or the inpatient ECG monitoring (44 cases). Patients with NVAF were older and had a shorter time window of onset to door. Coronary artery disease and heart failure are more prevalent in NVAF patients, although less of these patients were current smokers. NVAF patients were likely to have higher blood pressure and creatinine and take anticoagulant agents. The medium of CHA2DS2-VASc score was higher in NVAF compared with non-AF patients (3.0 versus 2.0) on admission. The number of vascular risk factors was not significantly different between patients with NVAF and those without, although 3 more risk factors were more likely to be recorded in the NVAF group (Fig. 2). The stroke etiology in NVAF group consisted of 71.6% (159/222) cardioembolic stroke and 28.4% (63/222) undetermined cause. However, 22.2% (1007/4531) large artery atherosclerosis (LAA) and 28.2% (1278/4531) small artery occlusion accounted for the half in the non-AF group.

**Table 1:** Baseline Characteristics of Patients Based on Atrial Fibrillation

Variables	Total	NVAF	Non-AF	*P* value
	(n = 4753 [100%])	(n = 222 [4.7%])	(n = 4531 [95.3%])	
Age, y	61.9 ± 11.1	71.1 ± 8.9	61.4 ± 11.0	< 0.001
Female	1413 (29.7)	69 (31.1)	1344 (29.7)	0.65
Medical history				
Current smoking	1563 (32.9)	43 (19.4)	1520 (33.5)	< 0.001
Hypertension	3005 (63.2)	138 (62.2)	2867 (63.3)	0.74
Diabetes mellitus	1114 (23.4)	41 (18.5)	1073 (23.7)	0.07
Dyslipidemia	381 (8.0)	19 (8.6)	362 (8.0)	0.76
Prior CAD	509 (10.7)	65 (29.3)	444 (9.8)	< 0.001
Heart failure	23 (0.5)	8 (3.6)	15 (0.3)	< 0.001
Prior Stroke/TIA	1073 (22.6)	49 (22.1)	1024 (22.6)	0.85
PVD	40 (0.8)	2 (0.9)	38 (0.8)	0.92
BMI	24.7 (22.9–26.7)	24.2 (22.0-26.4)	24.7 (22.9–26.7)	0.06
Baseline NIHSS	2.0 (1.0–4.0)	2.0 (1.0–4.0)	2.0 (1.0–4.0)	0.37
0	591 (12.4)	28 (12.6)	563 (12.4)	
1	965 (20.3)	35 (15.8)	930 (20.5)	
2	1038 (21.8)	58 (26.1)	980 (21.6)	
3	851 (17.9)	31 (14.0)	820 (18.1)	
4	774 (16.3)	44 (19.8)	730 (16.1)	
5	534 (11.2)	26 (11.7)	508 (11.2)	
CHA2DS2-VASc	2.0 (1.0–3.0)	3.0 (2.0–4.0)	2.0 (1.0–3.0)	< 0.001
Time measure				
Onset to door, hours	5.9 (2.4–12.4)	3.7 (1.6–7.2)	6.0 (2.5–12.6)	< 0.001
≤3 h	1513 (31.8)	97 (43.7)	1416 (31.3)	
3-4.5 h	503 (10.6)	33 (14.9)	470 (10.4)	
>4.5 h	2737 (57.6)	92 (41.4)	2645 (58.4)	
Length of stay	12.0 (10.0–15.0)	13.0 (10.0–15.0)	12.0 (10.0–14.0)	0.10
Laboratory				
Baseline blood glucose, mmol/L	5.5 (4.9-7.0)	5.5 (4.9–6.5)	5.5 (4.9-7.0)	0.21
Serum creatinine, µmol/L	69.0 (58.0–81.0)	75.8 (63.3–92.0)	69.0 (58.0–80.0)	< 0.001
Serum ALT, U/L	18.0 (13.0–25.0)	17.0 (13.0–25.0)	18.0 (13.0–25.0)	0.24
SBP at admission, mmHg	150.0 (136.5–165.0)	147.0 (134.0-164.0)	150.0 (137.0-165.0)	0.001
DBP at admission, mmHg	87.0 (80.0–96.0)	83.5 (77.0–91.0)	87.0 (80.0-96.5)	< 0.001
Medication history				
Antiplatelet	823 (17.3)	76 (34.2)	747 (16.5)	< 0.001
Anticoagulant	29 (0.6)	15 (6.8)	14 (0.3)	< 0.001
In-hospital treatment				
Thrombolysis	647 (13.6)	38 (17.1)	609 (13.4)	0.12
EVT	8 (0.2)	1 (0.5)	7 (0.2)	0.29
Antiplatelet	4640 (97.6)	192 (86.5)	4448 (98.2)	< 0.001
Anticoagulant	402 (8.5)	104 (46.8)	298 (6.6)	< 0.001
Warfarin	74 (1.6)	60 (27.0)	14 (0.3)	< 0.001
DOACs				
Rivaroxaban	4 (0.1)	2 (0.9)	2 (0.0)	< 0.001
Dabigatran	16 (0.3)	14 (6.3)	2 (0.0)	< 0.001
Antihypertensive agents	2251 (47.4)	106 (47.7)	2145 (47.3)	0.91
Lipid-lowering agents	4577 (96.3)	213 (95.9)	4364 (96.3)	0.78
Glucose-lowering agents	1202 (25.3)	38 (17.1)	1164 (25.7)	0.004

NVAF indicates nonvalvular atrial fibrillation; CAD, coronary artery disease; TIA, transient ischemic attack; PVD, peripheral vascular disease; BMI, body mass index; NIHSS, National Institutes of Health Stroke Scale; CHA_2_DS_2_-VASc, congestive heart failure, hypertension, age ≥ 75 years, diabetes, stroke/transient ischemic attack/thromboembolism, vascular disease, age 65 to 74 years, and sex category; ALT, alanine aminotransferase; SBP, systolic blood pressure; DBP, diastolic blood pressure; EVT, endovascular treatment; DOAC, direct oral anticoagulant

### Cardiovascular mortality

At 1-year follow-up, NVAF was associated with cardiovascular mortality compared with non-AF before adjusting potential covariates (HR, 4.13; 95% CI, 1.84 to 9.31; *P* < 0.001). Similar effects were detected for cardiovascular mortality after adjusting age, current smoking, diabetes mellitus, prior CAD, heart failure, BMI, onset to door time, blood creatinine, admission SBP, admission DBP, medication history of antiplatelet and anticoagulant, treatment of antiplatelet, anticoagulant, and glucose-lowering agents (HR, 4.66; 95% CI, 1.79 to 12.15; *P* = 0.001) (Table 2, Supplementary Fig. 1).

**Table 2:** Outcomes Among Acute Minor Ischemic Stroke Patients With and Without AF

Outcomes	N(%)		Unadjusted		Adjusted	
	NVAF	Non-AF	HR (95% CI)	P value	HR (95% CI)	P value
In-hospital						
All-cause mortality	0 (0)	3 (0.07)	-		-	
Cardiovascular mortality	0 (0)	3 (0.07)	-			-
Recurrent ischemic stroke	8 (3.6)	226 (4.99)	0.73 (0.36,1.48)	0.38	0.45 (0.19,1.05)	0.07
Hemorrhagic stroke	1 (0.45)	6 (0.13)	2.46 (0.22,27.27)	0.46	-	
3 months						
All-cause mortality	2 (0.9)	29 (0.64)	1.41 (0.34,5.90)	0.64	1.11 (0.24,5.10)	0.90
Cardiovascular mortality	2 (0.9)	21 (0.46)	1.94 (0.46,8.29)	0.37	1.82 (0.37,8.96)	0.46
Recurrent ischemic stroke	10 (4.5)	293 (6.47)	0.69 (0.37,1.30)	0.25	0.33 (0.16,0.68)	0.003
Hemorrhagic stroke	3 (1.35)	18 (0.4)	3.41 (1.00,11.57)	0.05	3.62 (0.69,19.01)	0.13
6 months						
All-cause mortality	4 (1.8)	44 (0.97)	1.85 (0.67,5.16)	0.24	1.12 (0.34,3.65)	0.86
Cardiovascular mortality	3 (1.35)	26 (0.57)	2.36 (0.71,7.78)	0.16	1.96 (0.49,7.82)	0.34
Recurrent ischemic stroke	15 (6.76)	339 (7.48)	0.89 (0.53,1.50)	0.67	0.49 (0.27,0.89)	0.02
Hemorrhagic stroke	3 (1.35)	24 (0.53)	2.56 (0.77,8.50)	0.12	2.82 (0.61,13.13)	0.19
1 year						
All-cause mortality	12 (5.41)	72 (1.59)	3.45 (1.87,6.35)	< 0.001	1.96 (0.92,4.15)	0.08
Cardiovascular mortality	7 (3.15)	35 (0.77)	4.13 (1.84,9.31)	< 0.001	4.66 (1.79,12.15)	0.001
Recurrent ischemic stroke	19 (8.56)	408 (9)	0.94 (0.60,1.50)	0.81	0.55 (0.32,0.94)	0.03
Hemorrhagic stroke	5 (2.25)	34 (0.75)	3.04 (1.19,7.77)	0.02	2.83 (0.83,9.61)	0.10

AF, indicates atrial fibrillation; NVAF, nonvalvular atrial fibrillation. Adjustment covariates includes age, current smoking, diabetes mellitus, prior CAD, heart failure, BMI, onset to door time, blood creatinine, admission SBP, admission DBP, medication history of antiplatelet and anticoagulant, treatment of antiplatelet, anticoagulant, and glucose-lowering agents

### All-cause mortality

Our analysis showed that all-cause mortality did not differ between NVAF and without AF groups at 3- and 6-month follow-up, whether univariate or multivariate analyses. At 1-year follow-up, NVAF was associated with all-cause mortality compared with non-AF before adjusting potential covariates (HR, 3.45; 95% CI, 1.87 to 6.35; *P* < 0.001). Nevertheless, after adjusting age, current smoking, diabetes mellitus, prior CAD, heart failure, BMI, onset to door time, blood creatinine, admission SBP, admission DBP, medication history of antiplatelet and anticoagulant, treatment of antiplatelet, anticoagulant, and glucose-lowering agents, no correlation was demonstrated between NVAF and all-cause mortality (HR, 1.96; 95% CI, 0.92 to 4.15; *P* = 0.08) (Table 2).

### Recurrent ischemic stroke

**Fig. 3:**
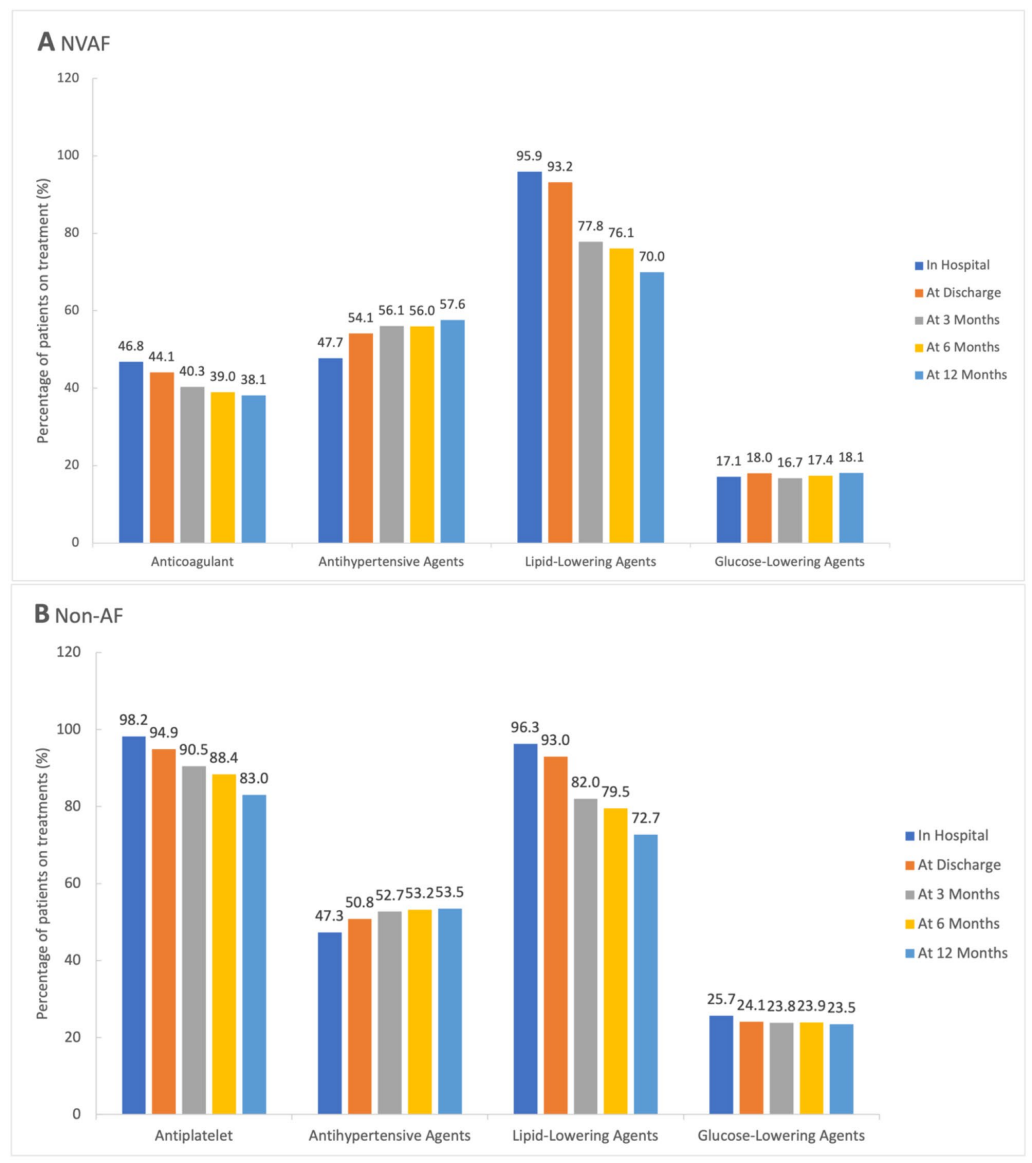
Secondary preventive medication at different follow-up points **A**, NVAF; **B**, Non-AF. AF indicates atrial fibrillation

About 40% of patients took anticoagulant agents in the NVAF group at follow-up term (Fig. 3). There was no statistical difference in regular antithrombotic therapy between the two groups. There was no difference in the in-hospital ischemic stroke recurrence rate between the two groups (HR, 0.45 [95% CI, 0.19 to 1.05] *P* = 0.07 at discharge). However, Patients with NVAF had a lower rate of recurrent ischemic stroke at 3-, 6- and 12-month follow-up (HR, 0.33 [95% CI, 0.16 to 0.68] *P* = 0.003 at 3 months; 0.49 [95% CI, 0.27 to 0.89] *P* = 0.02 at 6 months; 0.55 [95% CI, 0.32 to 0.94] *P* = 0.03 at 12 months, respectively) (Table 2, Supplementary Fig. 1).

### Recurrent hemorrhagic stroke

The absolute proportions of recurrent hemorrhagic stroke in the NVAF group were higher than that in the non-AF group at short-, medium-, and long-term follow-up. However, NVAF was not associated with recurrent hemorrhagic stroke at discharge, 3- and 6-month both in univariate and multivariate analyses. Only at 12-month follow-up NVAF was found to be associated with recurrent hemorrhagic stroke in univariate analysis (HR, 3.04; 95% CI, 1.19 to 7.77; *P* = 0.02), and in multivariate analysis, this correlation was not detected (HR, 2.83; 95% CI, 0.83 to 9.61; *P* = 0.10) (Table 2).

## Discussion

Our study found that acute minor ischemic stroke with NVAF was associated with cardiovascular mortality during the follow-up term extended. In contrast, NVAF was negatively associated with recurrent ischemic stroke compared with non-AF.

Although all-cause mortality was similar, NVAF was associated with cardiovascular mortality over time compared with non-AF. It might arise from the fact patients with AF usually had increased burden of multimorbidity, especially CAD and heart failure [14, 15], which means more severe heart damage and carried higher risk of cardiac death. Our findings were consistent with previous studies [6, 16–22]. One prior study from the Framingham Heart Study cohort has shown that AF could confer a 1.5- to 1.9-fold mortality risk [16]. Some hospital-based studies also verified that AF was associated with in-hospital, 3-, 6- and 12-month mortality in patients with ischemic stroke [6, 17–22]. However, most of these studies focused on moderate and severe stroke [23]. Another studies also showed AF was an independently predictor of death in patients with minor stroke including transient ischemic attack [24, 25]. Cardiometabolic comorbidity including AF could increase 5-year death by 1.89-fold for patients with a first transient ischemic attack [26].

Our study documented NVAF was associated with less recurrent ischemic stroke compared with non-AF. It is important to note that purely in terms of absolute recurrent rate, the patients with NVAF had an ischemic stroke recurrent rate of 8.56% at 1-year follow-up, which was not low compared with previous studies. Patients with NVAF without anticoagulants from Korea, Europe, and Denmark, who had the same CHA_2_DS_2_-VASc score of 3, had ischemic stroke incident rates (per 100 person-years) of 2.88, 4.77, and 4.1, respectively [27]. The relatively low stroke recurrence rate in patients with NVAF in our study may be attributed to two factors. Firstly, the use of anticoagulant medication has increased over time, as shown by the analysis of complete data of CNSR-III. The 12-month persistence of anticoagulants increased from 8.0% to 2007 to 2008 to 34.5% in 2015 to 2018. This increase in anticoagulant use may explain why AF was no longer an independent risk factor which associated with stroke recurrence in CNSR-III between 2015 and 2018 [28]. Secondly, 98 cases of NVAF were diagnosed by admission or hospitalization electrocardiogram, which can be classified as AF detected after stroke (AFDAS). Clinical evidence supports post-stroke neurogenic mechanisms in patients with AFDAS, and the paradigm of neurogenic AFDAS would be a low-burden paroxysmal AF occurring early after a stroke [29]. Several clinical studies suggested that the stroke risk associated with paroxysmal AF was lower than that of persistent AF [27, 30, 31].

In the present analysis, there was no statistical difference in recurrent hemorrhagic stroke between NVAF and non-AF groups. This is inconsistent with other studies. Studies from Chinese and Austrian stroke registries showed patients with AF suffered more frequently hemorrhagic transformation [20, 22]. A possible explanation for this discrepancy might be these studies enrolled stroke patients with much higher NIHSS scores associated with more severe brain injury and a high risk for hemorrhagic events. These findings underline the necessity that one more accurate stroke risk prediction model for AF patients rather than the current widely used CHA2DS2-VASc score are needed. All the AFDAS patients should be treated with anticoagulation based on CHA2DS2-VASc score, which may cause them at a higher risk of bleeding with little benefit of stroke prevention.

This study has several limitations. First, it was a cohort study and might have potential biases. Nevertheless, the CNSR-III was a large-scale nationwide prospective registry with broad representative of clinical practice for patients with minor stroke and NVAF. Second, the small number of patients in NVAF group and lower incidence of events limited power to estimate the effect size in hemorrhagic stroke between groups. Third, we did not compare KAF and AFDAS effects on prognosis due to limited sample, which is still waiting for further investigations.

## Conclusions

NVAF was associated with cardiovascular mortality over time and carried a lower risk of recurrent ischemic stroke compared with non-AF in minor ischemic stroke. A more accurate stroke risk prediction model for NVAF is warranted for optimal patient care strategies.

### Electronic supplementary material

Below is the link to the electronic supplementary material.

Supplementary Material 1

## Data Availability

The data and analytic methods of this study are available from the corresponding authors upon reasonable request and with approval from the Third China National Stroke Registry (CNSR-III) investigators.

## List of Abbreviations

NVAF: Nonvalvular atrial fibrillation
CHANCE: Clopidogrel in High-Risk Patients with Acute Nondisabling Cerebrovascular Events
POINT: Platelet-Oriented Inhibition in New TIA and Minor Ischemic Stroke
CNSR-III: The Third China National Stroke Registry
AIS: Acute ischemic stroke
TIA: Transient ischemic attack
NIHSS: National Institutes of Health Stroke Scale
mRS: Modified Rankin Scale
