# Supplementary material for: Short- and long-term outcomes of patients with minor stroke and nonvalvular atrial fibrillation
Source: BMC Neurol. 2023 Nov 20;23:410. doi: 10.1186/s12883-023-03457-3 (PMC10658860; doi:10.1186/s12883-023-03457-3)
Supplement: Supplementary file 1 — Supplementary Material 1 [file 12883_2023_3457_MOESM1_ESM.docx]

**Title Page**

Short- and Long-Term Outcomes of Patients with Minor Stroke and Nonvalvular Atrial Fibrillation

Chunmiao Duan, PhD^1,2,3^*; Shang Wang, MD, PhD^4^*; Yunyun Xiong, MD, PhD^1,2,5^; Hong qiu Gu, PhD^2,6^; Kaixuan Yang, PhD^2,6^; Xing-Quan Zhao, MD, PhD^1,2^; Xia Meng, MD, PhD^2^; Yongjun Wang, MD, PhD^1,2,6,7^

^1^Vascular Neurology, Department of Neurology, Beijing Tiantan Hospital, Capital Medical University, Beijing, China

^2^China National Clinical Research Center for Neurological Diseases, Beijing, China

^3^Department of Neurology, Beijing Daxing Teaching Hospital, Capital Medical University, Beijing, China

^4^Neurocardiology Center, Department of Neurology, Beijing Tiantan Hospital, Capital Medical University, Beijing, China

^5^Chinese Institute for Brain Research, Beijing, China

^6^National Center for Healthcare Quality Management in Neurological Diseases, Beijing, China

^7^Center for Stroke, Beijing Institute for Brain Disorders, Beijing, China

*Chunmiao Duan and Shang Wang contributed equally.

**Supplementary material**

**Supplementary Figure 1. Kaplan-Meier curve for the recurrent stroke and death**

**
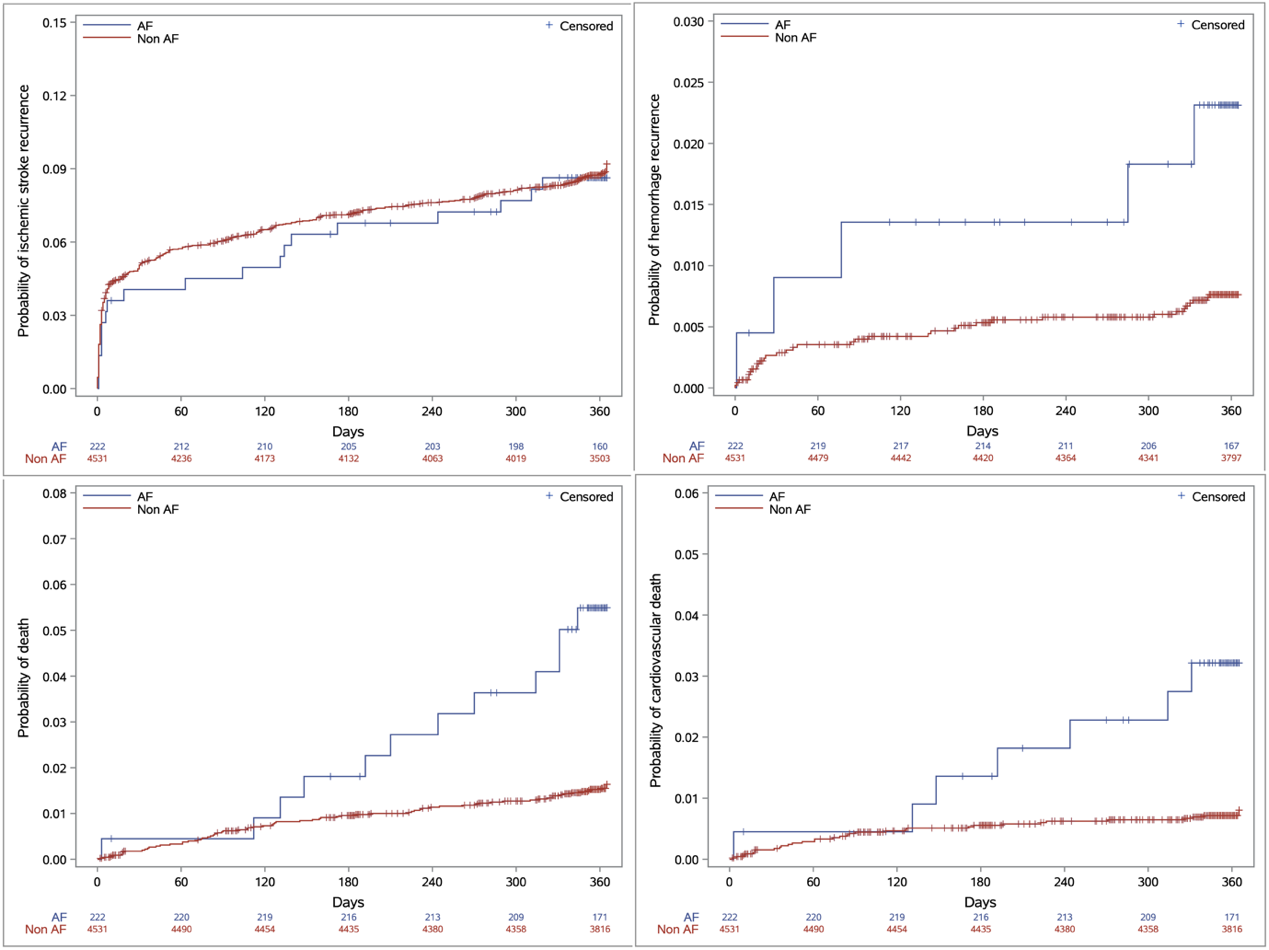
**
